# Baseline platelet count may predict short-term functional outcome of cerebral infarction

**DOI:** 10.1186/s12883-022-02845-5

**Published:** 2022-08-25

**Authors:** Kazo Kanazawa, Nobukazu Miyamoto, Kenichiro Hira, Chikage Kijima, Yuji Ueno, Nobutaka Hattori

**Affiliations:** Department of Neurology, Juntendo University School of Medicine, 3-1-3 Hongo, Bunkyo-ku, Tokyo, 113-0033 Japan

**Keywords:** Platelet count, Cerebral infarction, Neurological deterioration, Early prognosis, Risk factors

## Abstract

**Background and aims:**

Platelets play an important role in homeostasis however, they have also been associated with increased mortality after myocardial infarction. In the present study, we investigated whether platelet count is associated with differences in the short-term prognosis at the time of hospital discharge and early neurological deterioration in ischemic stroke patients.

**Methods:**

Patients with ischemic stroke were enrolled from among 661 cerebrovascular disease patients admitted between January 2018 and December 2020. Patients who received hyperacute treatment, had a pre-onset modified Rankin scale (mRS) ≥ 3, transient ischemic attack, or active malignant disease were excluded. The platelet count was divided into quartiles (Q1-4) according to the number of patients, and the relationship between platelet count and prognosis was assessed using multivariable analysis.

**Results:**

In total, 385 patients were included in the study. Regarding the functional outcome by platelet count, there was a significant increase in mRS ≥ 3 at discharge in the Q4 (range: 243–1327 × 10^9^/L, *p* = 0.013, ORs: 1.674, 95%CI: 1.253–6.681) group compared to the Q3 (range: 205–242 × 10^9^/L) group even after adjusting for factors with *P* < 0.2 in univariate analysis. Furthermore, the frequency of neurological deterioration (NIHSS ≥ 4) within 1 week was significantly lower in the Q3 group than in the Q1 (range; 19–173 × 10^9^/L) and Q4 groups even after adjustment (Q1; *p* = 0.020 ORs: 6.634, 95%CI: 1.352–32.557, Q4; *p* = 0.007 ORs: 8.765, 95%CI: 1.827–42.035).

**Conclusion:**

Platelet count at onset may affect the prognosis of cerebral infarction and early neurological deterioration. This study may help clarify the pathogenesis of cerebral infarction to improve prognosis.

## Introduction

Platelet count measurements are frequently performed in clinical practice to assess bleeding risk and thrombosis. Inter-individual differences in platelet counts are highly variable [1]; however, platelet counts in healthy individuals are usually stable and exhibit little intra-individual variability over time [2]. A platelet count of approximately 50–100 × 10^9^/L is generally sufficient to maintain vascular integrity and prevent spontaneous bleeding. However, a normal platelet count is 150–400 × 10^9^/L, suggesting that platelets have another functions (e.g. homeostasis maintenance) other than hemostasis [3, 4]. Consistent with this, a series of reports suggested that platelets play a role in physiological responses such as angiogenesis, fibrogenesis, and immune responses [5–7]. Therefore, platelets not only reflect underlying disease, but platelet function may also influence disease morbidity and mortality. An abnormal platelet count is a poor prognostic factor in some patient groups such as critically ill patients [8] and cancer patients [9, 10]. Furthermore, platelet counts outside the reference range are associated with mortality in the elderly and general population [11–13]. Recently, a U-shaped relationship between platelet count and mortality in the elderly population was reported [14]. Platelet counts have previously been linked to cause-specific mortality from cancer and cardiovascular disease [14]. In the field of cerebral infarction, platelet-related factors, such as CD40 ligand, monocyte-platelet aggregate formation and platelet-derived von Willebrand factor, are correlated with poor functional prognosis in stroke patient serum study and basic animal study [15, 16]. Du et al. [17] reported that elevated platelet counts increase the risk of ischemic stroke. In addition, Ye et al. showed that median platelet count group showed good stroke prognosis with long-term rehabilitation, but this was not a simple linear correlation [18]. However, reports on the relationship between platelet count and prognosis and neurological symptom exacerbation after stroke are limited, and the relevance is unclear. In this study, we analyzed whether platelet count is associated with differences in the short-term prognosis at the time of hospital discharge and early neurological deterioration in cerebral infarction.

## Patients and methods

### Patients

The study population comprised 661 Japanese patients who were admitted to our neurology department at Juntendo University Hospital within 2 days of acute stroke between January 2018 and December 2020. Exclusion criteria were: diagnosed with transient ischemic attack (TIA), underwent intravenous tissue plasminogen activator/endovascular treatment or malignancy treatment, or had a modified Rankin scale (mRS) ≥ 3. When the patients recovered to the functional stage from the assistance-free stage, they were discharged from the hospital to home. The endpoint of the trial was discharge from the hospital, and the study endpoints were the rate of mRS 3–6 at discharge and early neurological deterioration within 1 week from onset.

### Background data and risk factors

We retrieved the following information from the medical records of each patient to evaluate the short-term prognosis at the time of hospital discharge and subsequent deterioration: 1) demographic data, 2) vital signs at presentation and laboratory findings on admission, 3) medications being taken upon admission, with particular attention paid to anti-platelets, anti-coagulants, anti-hypertensives, and statins, 4) vascular risk factors for stroke such as hypertension (HT; systolic blood pressure [BP] > 140 mmHg, diastolic BP > 90 mmHg, or drug treatment for HT), dyslipidemia (DL; low-density lipoprotein [LDL] cholesterol level of > 140 mg/dl, high-density lipoprotein [HDL]-cholesterol level of < 40 mg/dl, triglyceride [TG] level of > 149 mg/dl, or drug treatment for DL), diabetes mellitus (DM; glycated hemoglobin [Hb_A1c_] level of > 6.8%, or drug treatment for DM), a cardioembolic source according to the Trial of Org 10,172 in Acute Stroke Treatment (TOAST) classification [19], TIA, and smoking history (as reported by the patient and their family), 5) stroke mechanism according to the TOAST criteria [19], and 6) the baseline National Institutes of Health Stroke Scale (NIHSS) score [20], as recorded by stroke-trained neurologists that were certified in the application of the NIHSS on admission, at 7 days after admission, and upon discharge. The deterioration of neurological findings was defined as worsening of the NIHSS score by ≥ 4 points within 1 week of admission to the hospital. Brain computed tomography (CT)/magnetic resonance imaging (MRI) and electrocardiography were performed in all patients. Brain MRI was conducted in all applicable patients. We diagnosed brain infarction by focal hyper-intensity that was judged not attributable to normal anisotropic diffusion or magnetic susceptibility artifact.

Briefly, according to Japanese stroke guidelines, thrombosis was treated with dual antiplatelet therapy (often use aspirin 200 mg/day and clopidogrel 75 mg), with edaravone and argatroban in acute phase. For cardiogenic embolism, we use edaravone in acute phase, and stated anticoagulant after day 2–5 from stroke onset. For embolic stroke of undetermined source, we treat with aspirin and edaravone.

### Ethical consideration and statistical analysis

The protocol of this retrospective study was approved by the Human Ethics Review Committee of Juntendo University School of Medicine. The data were analyzed with SPSS 17.0 (SAS Institute Inc., Cary, NC) and are expressed as mean ± SD values. All statistical analyses were performed using χ^2^ test for categorical variables and Kruskal Wallis test for non-parametric analyses. The platelet count was divided into quartiles and used in the multiple logistic regression analysis to estimate the relationship. Variables with a *P* value < 0.2 on univariate analysis were entered into multiple logistic regression analysis. *p*-values of < 0.05 were considered significant.

## Results

**Fig. 1:**
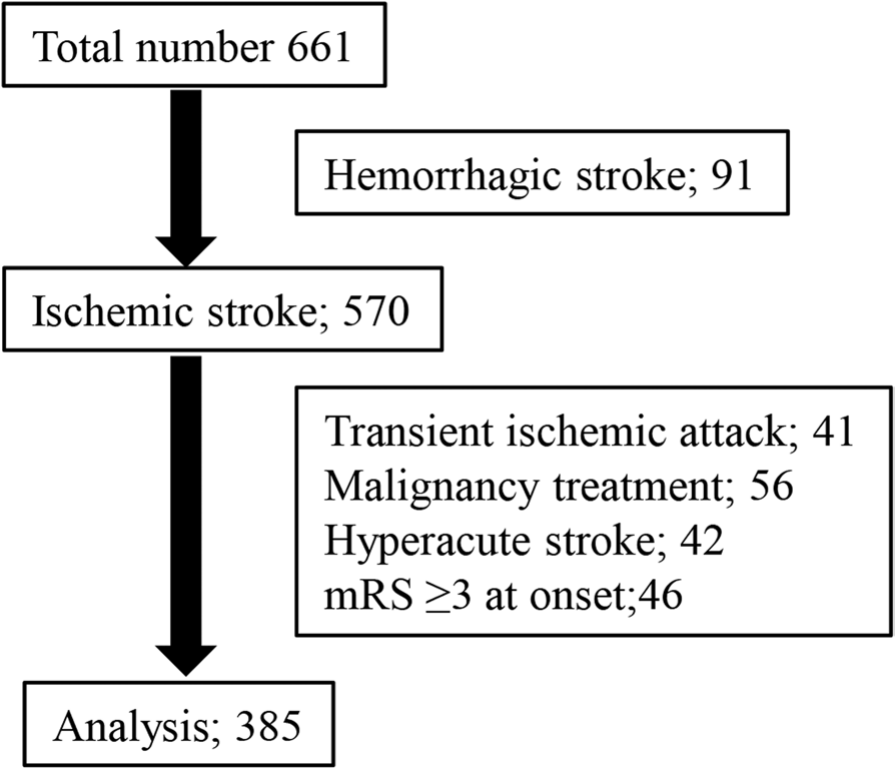
Flow chart describing enrollment of patients with stroke in the present study

A total of 385 patients were enrolled in this study after excluding patients who were diagnosed with hemorrhagic stroke (*n* = 91), TIA (*n* = 41), undergoing malignancy treatment (*n* = 56), mRS ≥ 3 (*n* = 46), or receiving intravenous tissue plasminogen activator treatment and endovascular treatment (*n* = 42) (Fig. 1). Based on the final diagnosis using TOAST criteria, the following stroke subtypes were confirmed: small-vessel occlusion (SVO, *n* = 50, 13.0%), large-artery atherosclerosis (LAS, *n* = 47, 12.2%), cardioembolism (CE, *n* = 107, 27.8%), and other determined etiology (branch atheromatous disease [BAD], *n* = 68, 7.3%). Early neurological deterioration of NIHSS ≥ 4 within 1 week from onset was noted in 35 patients (9.1%).

The patient background by platelet count quartiles is shown in Table 1 (range × 10^9^/L; Q1 19–173, Q2 174–204, Q3 205–242, Q4 243–1327, mean ± SD × 10^9^/L: overall mean 215 ± 86, Q1 = 138 ± 31, Q2 = 190 ± 9, Q3 = 223 ± 11, Q4 = 307 ± 117). Between quartiles, body mass index, smoking habit, and systolic blood pressure significantly differed (p < 0.05), but age, sex, HT, DM, DL, ischemic heart disease (IHD), atrial fibrillation (Af), history of cerebral infarction, and NIHSS on onset were not different. Stroke subtypes (SVO, LAS, CE, and BAD) and premedication from stroke onset did not differ, except for ARB medication (*p* < 0.05). Regarding laboratory findings (Table 2), white blood cell count, D-dimer, and N-terminal pro-brain natriuretic peptide (NT-proBNP) levels significantly differed between quartiles (*p* < 0.05). However, there were no differences in eGFR, UA, HDL, LDL, TG, blood sugar, or Hb_A1c_ at admission. In patient outcome (Table 3), mRS ≥ 3 (mRS3-6) at discharge and early neurological deterioration significantly differed between platelet quartiles (*p* < 0.05). However, antiplatelet therapy and anticoagulant therapy for secondary prevention were not differed in platelet query groups.

**Table 1:** Patient background data

Platelet quartiles platelet count range (× 10^9^/L)	Q1 (96) 138 ± 31 19–173		Q2 (96) 190 ± 9 174–204		Q3 (96) 223 ± 11 205–242		Q4 (97) 307 ± 117 243–1327		*P*-value
	N	%	N	%	N	%	N	%	
sex (male)	60	62.5	65	67.7	64	66.7	56	57.7	0.480
age	71.8 ± 14.7		69.7 ± 15.0		69.0 ± 13.8		67.1 ± 14.1		0.071
BMI	22.9 ± 3.7		23.7 ± 3.21		24.5 ± 3.5		22.8 ± 3.3		0.004
smoking habit	13	13.5	25	26.0	13	13.5	29	29.9	0.006
hypertension	67	69.8	69	71.9	81	84.4	72	74.2	0.920
sBP	147.5 ± 28.5		152.5 ± 25.1		160.5 ± 29.0		155.1 ± 32.2		0.025
dBP	83.6 ± 17.7		85.6 ± 14.5		90.0 ± 19.7		89.2 ± 19.4		0.205
diabetes	44	45.8	43	44.8	37	38.5	34	35.1	0.368
dyslipidemia	71	74.0	73	76.0	70	72.9	70	72.2	0.935
IHD	10	10.4	10	10.4	10	10.4	9	9.3	0.944
Af	28	29.2	24	25.0	20	20.8	22	22.7	0.569
history of CI	13	13.5	13	13.5	18	18.8	16	16.5	0.705
NIHSS ≥ 8	15	15.6	10	10.4	13	13.5	9	9.3	0.518
NIHSS	4.8		3.2		3.9		3.5		0.940
Stroke subtype									
SVO	6	6.3	16	16.7	15	15.6	13	13.4	0.132
LAS	16	16.7	6	6.3	10	10.4	15	15.5	0.102
CE	29	30.2	27	28.1	25	26.0	26	26.8	0.924
BAD	6	6.3	10	10.4	7	7.3	5	5.2	0.532
Pre-medication									
CaB	21	21.9	25	26.0	37	38.5	31	32.0	0.064
ARB	16	16.7	23	24.0	32	33.3	20	20.6	0.044
ACE-I	8	8.3	2	2.1	3	3.1	3	3.1	0.123
diuretics	15	15.6	6	6.3	6	6.3	4	4.1	0.016
statins	28	29.2	33	34.4	30	31.3	19	19.6	0.123
DPP4/GLP1	19	19.8	14	14.6	15	15.6	11	11.3	0.438
anticoagulant	17	17.7	20	20.8	9	9.4	13	13.4	0.135
antiplatelet	23	24.0	24	25.0	21	21.9	18	18.6	0.718

Values are expressed as mean ± SD except for NIHSS which is expressed as the median

*BMI* body mass index, *sBP* systolic blood pressure, *dBP* diastolic blood pressure, *IHD* ischemic heart disease, *Af* atrial fibrillation, *history of CI* history of cerebral infarction, *NIHSS* National Institutes of Health Stroke Scale, *SVO* small-vessel occlusion, *LAS* large-artery atherosclerosis, *CE* cardiogenic embolism, *BAD* branch atheromatous disease, *CaB* calcium channel blocker, *ARB* angiotensin II receptor blocker, *ACE-I* angiotensin-converting-enzyme inhibitor, *DPP4* dipeptidyl peptidase 4 inhibitor, *GLP1* glucagon-like peptide-1

**Table 2:** Laboratory data on admission

Platelet quartiles platelet count range (× 10^9^/L)	Q1 (96) 138 ± 31 19–173	Q2 (96) 190 ± 9 174–204	Q3 (96) 223 ± 11 205–242	Q4 (97) 307 ± 117 243–1327	*P*-value
WBC	6336 ± 2141	6861 ± 2648	7368 ± 2380	7758 ± 2840	0.001
Hb	13.44 ± 2.18	14.09 ± 1.96	14.02 ± 2.04	13.49 ± 2.13	0.059
hsCRP	1.458 ± 4.475	0.501 ± 1.425	0.764 ± 2.031	1.260 ± 3.744	0.284
PT-INR	1.07 ± 0.17	1.25 ± 1.32	1.04 ± 0.12	1.08 ± 0.34	0.103
D-dimer	3.40 ± 3.65	2.21 ± 2.46	2.25 ± 2.25	2.32 ± 2.39	0.015
eGFR	64.1 ± 27.1	66.1 ± 22.3	63.0 ± 24.0	72.4 ± 26.5	0.100
UA	5.46 ± 1.58	5.56 ± 1.44	5.81 ± 1.60	1.58 ± 1.58	0.350
HDL	51.4 ± 15.0	53.1 ± 13.5	51.5 ± 15.1	54.5 ± 17.5	0.404
LDL	110.4 ± 40.0	112.3 ± 39.3	116.2 ± 31.8	122.9 ± 48.6	0.163
TG	136.9 ± 86.5	138.4 ± 93.0	145.8 ± 106.6	138.6 ± 94.2	0.981
blood sugar	144.5 ± 72.8	138.7 ± 60.6	131.5 ± 43.9	126.6 ± 51.9	0.287
Hb_A1c_	6.67 ± 1.86	6.57 ± 1.50	6.40 ± 1.29	6.38 ± 1.47	0.365
NTproBNP	2233.4 ± 4911.3	902.5 ± 2551.8	1594.7 ± 4967.2	1122.17 ± 5881.3	0.004

Values are expressed as mean ± SD

*hsCRP* high-sensitivity C-reactive protein, *PT-INR* prothrombin time-international normalized ratio, UA uric acid, *HDL* high-density lipoprotein, *LDL* low-density lipoprotein, *TG* triglyceride, *NT-proBNP* N terminal pro-brain natriuretic peptide

**Table 3:** Patient outcomes and anti-platelet/coagulant therapy for secondary prevention at discharge in platelet quartiles

Platelet quartiles platelet count range (× 10^9^/L)	Q1 (96) 138 ± 31 19–173		Q2 (96) 190 ± 9 174–204		Q3 (96) 223 ± 11 205–242		Q4 (97) 307 ± 117 243–1327		*P*-value
	N	%	N	%	N	%	N	%	
mRS3-6	22	22.9	10	10.4	10	10.4	25	25.7	0.004
deterioration	12	12.5	7	7.3	2	2.1	14	14.4	0.013
Medication for secondary prevention									
anticoagulant	42	43.7	40	41.6	34	35.4	39	40.2	0.681
antiplatelet	55	57.2	56	58.3	53	55.2	51	52.5	0.859

mRS3-6, modified Rankin scale ≥ 3; neurological deterioration was defined as NIHSS ≥ 4

To investigate the relationship between platelet count and patient outcome, we analyzed the patient background factors that were relevant to mRS ≥ 3 at discharge and early neurological deterioration. Univariate analysis revealed mRS ≥ 3 (Table 4), sex, age, smoking habit, dyslipidemia, Af, NIHSS ≥ 8, and WBC count were significant (p < 0.2). In stroke subtype, SVO and CE were significant (*p* < 0.2). We previously reported a good prognosis in patients taking angiotensin II receptor blocker (ARB) medication, and another report demonstrated that factors [21], such as statins and dipeptidyl peptidase 4 inhibitor/glucagon-like peptide-1 (DPP4/GLP-1) medication, were associated with prognosis [22]. Therefore, we analyzed premedication factors. Among premedication factors, ACE-I, statin, and diuretics were significant (*p* < 0.2). Likewise, early neurological deterioration was related (p < 0.2) to the factors of NIHSS ≥ 8, WBC count, Hb count, SVO, LAS, BAD, and anticoagulant premedication in univariate analysis (Table 5).

**Table 4:** Univariate analysis of mRS at discharge

	mRS0-2 (318)		mRS3-6 (67)		*P*-value
	N	%	N	%	
sex (male)	208	65.4	37	55.2	**0.125**
Age	67.8 ± 14.2		77.18 ± 13.4		** < 0.001**
BMI	22.6 ± 3.50		22.58 ± 3.50		0.380
smoking habit	68	21.8	12	17.9	0.620
hypertension	236	74.2	53	79.1	0.441
diabetes	134	42.1	24	35.8	0.412
dyslipidemia	241	75.7	43	64.1	**0.066**
IHD	30	9.4	8	11.9	0.504
Af	68	21.3	26	38.8	**0.004**
history of CI	49	15.4	11	16.4	0.853
NIHSS ≥ 8	18	15.6	29	43.2	** < 0.001**
WBC	6940 ± 2510		7761 ± 2732		**0.017**
Hb	13.7 ± 2.07		13.6 ± 2.22		0.649
Stroke subtype					
SVO	48	15.0	2	2.9	**0.005**
LAS	36	11.3	11	16.4	0.303
CE	78	24.5	29	43.2	**0.003**
BAD	22	6.9	6	8.9	0.604
Pre-medication					
CaB	92	28.9	22	32.8	0.557
ARB	75	23.5	16	23.8	0.998
ACE-I	10	3.1	6	8.9	**0.042**
diuretics	21	6.6	10	14.9	**0.044**
statins	97	30.5	13	19.4	**0.075**
DPP4/GLP1	52	16.3	7	10.4	0.266
anticoagulant	50	15.7	8	11.9	0.713
antiplatelet	70	22.1	16	23.8	0.748

Values are expressed as the mean ± SD. Bold *p*-values = *p* < 0.2

*BMI* body mass index, *IHD* ischemic heart disease, *Af* atrial fibrillation, history of CI history of cerebral infarction, *NIHSS* National Institutes of Health Stroke Scale, *SVO* small-vessel occlusion, *LAS* large-artery atherosclerosis, *CE* cardiogenic embolism, *BAD* branch atheromatous disease, *CaB* calcium channel blocker, *ARB* angiotensin II receptor blocker, *ACE-I* angiotensin-converting enzyme inhibitor, *DPP4* dipeptidyl peptidase 4 inhibitor, *GLP1* glucagon-like peptide-1

**Table 5:** Univariate analysis on neurological deterioration within 7 days from onset

Neurological deterioration	- (350)		+ (35)		*P*-value
	N	%	N	%	
sex (male)	224	64.0	21	60.0	0.713
age	69.0 ± 14.3		73.7 ± 15.4		0.318
BMI	23.5 ± 3.43		23.6 ± 4.33		0.220
smoking habit	72	20.5	8	22.8	0.827
hypertension	264	75.4	25	71.4	0.682
diabetes	147	42.0	11	31.4	0.280
dyslipidemia	259	74.0	25	71.4	0.693
IHD	37	10.45	1	2.8	0.231
Af	84	24.0	10	28.5	0.540
history of CI	49	14.0	11	31.4	0.853
NIHSS ≥ 8	37	10.5	10	28.5	**0.005**
WBC	7048 ± 2555		7452 ± 2674		**0.181**
Hb	13.8 ± 2.07		13.2 ± 2.27		**0.161**
Stroke subtype					
SVO	50	14.2	0	0	**0.014**
LAS	37	10.5	10	28.5	**0.005**
CE	98	28.0	9	25.7	0.846
BAD	21	6.0	7	20.0	**0.008**
Pre-medication					
CaB	103	29.4	11	31.4	0.847
ARB	86	24.5	5	14.2	0.213
ACE-I	14	4.0	2	5.7	0.648
diuretics	28	8.0	3	8.5	0.753
statins	101	28.8	9	25.7	0.845
DPP4/GLP1	56	16.0	3	8.5	0.328
anticoagulant	57	16.28	2	5.7	**0.137**
antiplatelet	75	21.4	11	31.4	0.201

*BMI* body mass index, *IHD* ischemic heart disease, *Af* atrial fibrillation, history of CI history of cerebral infarction, *NIHSS* National Institutes of Health Stroke Scale, *SVO* small-vessel occlusion, *LAS* large-artery atherosclerosis, *CE* cardiogenic embolism, *BAD* branch atheromatous disease, *CaB* calcium channel blocker, *ARB* angiotensin II receptor blocker, *ACE-I* angiotensin-converting enzyme inhibitor, *DPP4* dipeptidyl peptidase 4 inhibitor, *GLP1* glucagon-like peptide-1

Values are expressed as the mean ± SD. Bold p-values = *p* < 0.2

We performed logistic regression analysis of factors related to mRS3-6 and deterioration between platelet count query groups (Table 6). Due to the U-shaped relationship between platelet count and prognosis [14], and mean platelet count being 215 × 10^9^/L, we set the reference group as Q3. Factors that showed *p* < 0.2 in the univariate analysis were entered into the multivariable analysis. Regarding mRS3-6, with the reference set as Q3, the Q4 group had a significantly poorer prognosis (*p* < 0.05), and the Q1 group tended to have poor prognosis (*p* = 0.079). In the case of early neurological deterioration, the Q1 and Q4 groups had a higher rate than the Q3 group (*p* < 0.05).

**Table 6:** Logistic regression analysis for mRS3-6 and neurological deterioration

mRS3-6				Neurological deterioration			
PltQ	P-value	Odds	95%CI	PltQ	P-value	Odds	95%CI
Q1	0.079	2.159	0.914–5.101	Q1	0.020	6.634	1.352–32.557
Q2	0.837	1.107	0.422–2.900	Q2	0.078	4.433	0.848–23.164
Q3		ref		Q3		ref	
Q4	0.013	1.674	1.253–6.681	Q4	0.007	8.765	1.827–42.035

PltQ, platelet quartiles; mRS3-6, modified Rankin scale ≥ 3

including factors with *p* < 0.2 by univariate analysis

mRS3-6; sex, dyslipidemia, CE, ACE premedication, diuretics premedication, statin premedication, WBC, Af, NIHSS > 8

deterioration; anticoagulant therapy, SVD, ATBI, BAD, WBC, Hb, NIHSS > 8

## Discussion

This study demonstrated the relationship between platelet count and ischemic stroke prognosis at discharge/early neurological deterioration. The Q3 group (range: 205–242 × 10^9^/L, mean ± SD: 223 ± 11 × 10^9^/L) exhibited a low rate of early neurological deterioration and good prognosis compared with the Q1 and Q4 groups.

Our study is consistent with previous studies. One cohort study reported that platelet counts at the upper end of the normal range (301–450 × 10^9^/L) were associated with the development of cardiovascular disease [23], and the risk of ischemic stroke, myocardial infarction, and peripheral vascular disease were found to increase with platelet counts over 251 × 10^9^/L. A cohort study of 1506 men reported an association between the risk of ischemic stroke and platelet counts in the upper normal range [24]. The relationship between platelet count and prognosis is gradually becoming one of the focal points of clinical research [13, 23]. An analysis based on 3229 subjects from the Chinese Acute Ischemic Stroke Antihypertensive Study suggested that platelet count, especially the decrease due to platelet consumption, is an important non-negligible indicator in the prognosis of ischemic stroke [25]. It is well known that platelet activation is central to the process of arterial thrombus formation at the site of vascular injury [26]. Hence, antiplatelet therapy in arterial thromboembolism has received much attention, as antiplatelet agents prevent cerebrovascular disorders and cardiovascular events [26]. In addition, cardiovascular mortality increases in individuals with high platelet counts, especially in men and the elderly [14]. As reflected in these findings, our study also showed high platelet count group often occur neurological deterioration and appeared poor prognosis, even in no differences in stroke severity at onset between the groups based on the NIHSS score.

We found that early neurological deterioration in the low-platelet group (Q1) was significantly higher than the normal group, without stroke severity at onset. On the other hand, D'Erasmo et al. indicated the difference in platelet counts between the acute and recovery phases of stroke was not significant [27]. Several study suggests decreased platelet count is considered to be one of the risk factors for cerebral infarction [28]. Therefore, it is speculated that patients with low platelet counts often present with more obvious residual dysfunction, often with early neurologic exacerbations. In a previous basic study using stroke-prone spontaneously hypertensive rats, a lower platelet count was a predictor of asymptomatic cerebral small vessel disease and symptomatic stroke [29]. Thus, both clinical reports and basic studies suggest that low and high platelet counts are associated with cerebral infarction.

Recently, Vinholt and colleagues concluded that platelet counts are associated with cardiovascular and cerebrovascular disease [23]. In general, cerebral infarction is closely related to platelet function, and thrombosis is the result of activation of platelets and the coagulation system. Thus, platelet count and function may have a significant impact on the occurrence and development of cerebral infarction [17]. Atherosclerosis is the pathologic basis of cerebral infarction, especially SVO, LAS, and BAD. As atherosclerosis progresses, endothelial cells in the vessel wall are damaged. As a result, the contact area with platelets increases, and thrombosis is more likely to be induced [29]. Platelets have also been shown to influence the effect of homocysteine on the prognosis of ischemic stroke. Increased homocysteine blood levels in patients with decreased platelet counts increase ischemic stroke mortality, but not in patients with normal to increased platelet counts [25]. Moreover, normal platelets play an important role in cell proliferation, chemotaxis, cell differentiation, and angiogenesis by releasing natural cytokines. The basic cytokines identified in platelets include transforming growth factor-β, platelet-derived growth factor, basic fibroblast growth factor, vascular endothelial growth factor, and endothelial cell growth factor [30]. Thus, it can be inferred that the factors that regulate the cellular environment produced by platelets affect the prognosis of cerebral infarction.

### Limitations

Our study has several limitations involving its non-random treatment allocation procedure and retrospective design. It must be emphasized that this study is exploratory; thus, we aimed to generate hypotheses, but not to test them. Second, antiplatelet and anticoagulant therapy were administered in combination with antihypertensive drugs, statins, and antidiabetic drugs in some patients. This treatment approach may have reduced the severity of stroke by reflecting more advanced medical measures and more aggressive risk factor reduction. Third, because the study was retrospective, information on duration of treatment and daily medication compliance was not available. Fourth, we were unable to determine how different treatment and imaging modalities for acute ischemic stroke influenced our results. However, as the patients in this study were treated for ischemic stroke according to Japanese stroke guidelines, the impact of such differences in treatment was estimated to be limited. Fifth, in generally, this type of analysis would be better to use fractional polynomials or restricted cubic splines. The main research question of this paper was the relationship between platelet count and prognosis, we selected categorical data for importance of U-shape curve. Further prospective randomized studies are needed to address these uncertainties.

## Conclusion

In conclusion, lower and higher platelet counts at onset may affect the prognosis of cerebral infarction. Our clinical study demonstrated a U-curve. Maintaining a healthy platelet count may improve the prognosis of cerebrovascular disease. This may improve our understanding of the pathophysiology to help improve the prognosis of stroke.

## Data Availability

The datasets used analyzed during the current study are available from the corresponding author on reasonable request.

## Abbreviations

TIA: Transient ischemic attack
mRS: Modified Rankin scale
HT: Hypertension
BP: Blood pressure
DL: Dyslipidemia
LDL: Low-density lipoprotein
HDL: High-density lipoprotein
TG: Triglyceride
DM: Diabetes mellitus
Hb_A1c_: Glycated hemoglobin
TOAST: Trial of Org 10,172 in Acute Stroke Treatment
NIHSS: National Institutes of Health Stroke Scale
CT: Computed tomography
MRI: Magnetic resonance imaging
SVO: Small vessel occlusion
LAS: Large-artery atherosclerosis
CE: Cardiogenic embolism
BAD: Branch atheromatous disease
IHD: Ischemic heart disease
Af: Atrial fibrillation
mRS ≥ 3: MRS3-6
NT-proBNP: N-terminal pro-brain natriuretic peptide
DPP4: Dipeptidyl peptidase 4 inhibitor
GLP1: Glucagon-like peptide-1
PRP: Platelet-rich plasma
BMI: Body mass index
sBP: Systolic blood pressure
dBP: Diastolic blood pressure
history of CI: History of cerebral infarction
CaB: Calcium channel blocker
ARB: Angiotensin II receptor blocker
ACE-I: Angiotensin-converting enzyme inhibitor
hsCRP: High-sensitivity C-reactive protein
PT-INR: Prothrombin time-international normalized ratio
UA: Uric acid
PltQ: Platelet quartiles
